# Cis-regulatory functions of overlapping HIF-1alpha/E-box/AP-1-like sequences of CD164

**DOI:** 10.1186/1471-2199-12-44

**Published:** 2011-10-14

**Authors:** Jingqun Tang, Zhaohui Luo, Guangqian Zhou, Chao Song, Fenglei Yu, Juanjuan Xiang, Gang Li

**Affiliations:** 1 Department of Cardiothoracic Surgery, Xiangya Second Hospital, Central South University, 139 Renmin Zhong Road, Changsha, Hunan, 410011, P.R. China; 2 Cancer Research Institute, Key Laboratory of Carcinogenesis and Cancer Invasion of Ministry of Education, Key Laboratory of Carcinogenesis of Ministry of Health, Central South University, 110 Xiangya Road, Changsha, Hunan, 410078, P.R. China; 3 Centre for Cancer Research and Cell Biology, School of Biomedical Sciences, Queen's University Belfast, Belfast, BT9 7BL, Northern Ireland, UK; 4 School of Medicine, Shenzhen University, Shenzhen, P.R. China; 5 The Li Ka Shing Institute of Health Sciences, Department of Orthopaedics & Traumatology, Faculty of Medicine, The Chinese University of Hong Kong, Prince of Wales Hospital, Shatin, NT, Hong Kong; 6 Stem Cell and Regeneration Program, School of Biomedical Sciences, The Chinese University of Hong Kong, Shatin, NT, Hong Kong, P.R. China

## Abstract

**Background:**

CD164 (also known as MGC-24v or endolyn) is a sialomucin which has been suggested to participate in regulating the proliferation, cell adhesion and differentiation of hematopoietic stem and progenitor cells. CD164 is also involved in the development of cancer. The functions of cis-regulatory elements of CD164 remain relatively unknown.

**Methods:**

In this study, we investigated the function of cis-regulatory elements within the promoter of CD164. We fused the 5'-flanking region of CD164 to a luciferase reporter vector. The minimal promoter region was confirmed by luciferase reporter assay. Using *in silico *analysis, we found the presence of one HIF-1alpha (HIF-1A) motif (5_-RCGTG-3_) overlapping E-box (CACGTG) and two AP-1-like binding sites (CGCTGTCCC, GTCTGTTG), one of which is also overlapped with HIF-1alpha sequence. Dual-luciferase assay was performed to examine the transcriptional activity of AP-1 and HIF-1alpha of CD164 promoter. Quantitative reverse transcriptase polymerase chain reaction (qRT-PCR) was performed to measure CD164 expression. Chromatin Immunoprecipitation was used to confirm the binding of HIF-1alpha and CD164.

**Results:**

Co-transfection of c-jun, HIF-1alpha and minimal promoter region construct demonstrated that c-jun and HIF-1alpha bound the CD164 promoter and promoted CD164 expression. Hypoxia treatment also led to the up-regulation of CD164 expression. The mutation of overlapping sequences resulted in the reduced expression of CD164 induced by HIF-1alpha. Chromatin Immunoprecipitation demonstrated that the HIF-1alpha bound the minimal promoter region.

**Conclusions:**

Determination of the optimal promoter region and transcription factors governing CD164 expression is useful in understanding CD164 functions. These results suggest that cis-regulatory elements of CD164 overlapping HIF-1alpha/E-box/AP-1-like sequences may play important regulatory roles.

## Background

CD164 (MGC-24v or endolyn) is a member of the sialomucin family, a mucin containing sialic acid, which is highly conserved in humans and other species [1-4]. The human CD164 gene is located on chromosome 6q21 [2,4-6]. CD164 was first identified on CD34^+ ^human hematopoietic progenitor cells and bone marrow stromal reticular cells [2,6,7]. CD164 has been implicated in adhesion, proliferation and differentiation of hematopoietic stem and progenitor cells [6,8]. CD164 was suggested to mediate the adhesion of CD34^+ ^haematopoietic progenitor cells to bone marrow stromal cells and SDF-1-induced binding to bone marrow endothelial cells [2,9,10]. CD164 is thought to regulate hematopoiesis by facilitating the adhesion of human CD34+ cells to bone marrow stroma [5]. Knocking down CD164 expression in Drosophila S2 cells increased the cell apoptosis rate [1]. CD164 also participated in the localization of prostate cancer cells to the bone marrow and has been identified new markers for acute lymphoblastic leukaemia [11,12]. Previous work also confirmed the roles of CD164 on the development of colorectal cancer [13]. CD164 gene expression is regulated by specific transcription factors which bind to the promoter region, regulating cell growth and differentiation. Our attention has recently turned to an investigation of the cis-regulatory elements of CD164. Determining the optimal promoter region and transcription factors governing CD164 expression is important in understanding CD164 functions.

In the present study, we proceeded to identify CD164 promoter and performed the functional analysis of cis-regulatory element on CD164 expression.

## Methods

### 1. Prediction of promoter

Promoter 2.0 Prediction Server was employed for prediction of promoter. As CD164 first exon begins on 10418(AL359711 CD164 gDNA), ATGtcgcggc, the 3192 bp sequence 5' upstream of TSS were analyzed by the this method.

### 2. Construction of plasmids

Human c-jun expression plasmid pCMV-2/c-jun, which contains the full-length coding region of human c-jun cDNA cloned in pCMV-2 vector and pCMV-2 empty vector, were gifts from Dr. Gavin P. Collett (Department of Surgery, The Cancer Centre, Queen's University Belfast, UK). Vector plasmids pCDNA3.1-HIF-1A(1-826) and pCDNA3.1 were kindly donated by Dr. Zhou (Department of Orthopaedics and Traumatology, The University of Hong Kong). Firefly luciferase reporter plasmid pGL3-Basic was purchased from Promega. For construction of 5'-deletions of CD164 promoter reporter plasmids, human genomic DNA was isolated using the FlexiGene DNA Kit (Qiagen), the CD164 promoter candidate fragments were amplified by polymerase chain reaction (PCR) from genomic DNA, and PGL3P4.2-4 fragment was synthesized by two complementary strand oligonucleotides. Fragments were cloned into the pCR2.1-TOPO vector (Invitrogen). Cloning of different fragments to pGL3-Basic (Promega), which is a vector carrying the firefly luciferase gene were performed after the restrictive enzyme cut. The restrictive endonucleases used for the cloning are shown in Table 1. The restriction endonucleases which were used predominantly were Sac I and XholI, except P1 fragment, because of an internal SacI site existing in fragments between P1 and P1.5. P1 segment was obtained by using the KpnI and XholI sites in pCR2.1-TOPO vector. Finally, all plasmids were confirmed by sequencing. The oligonucleotides used for PCR primers are shown in Table 1.

**Table 1:** The oligonucleotides used for PCR primers

	Forward	Reverse	Restriction sites		Amplified DNA Fragment Length (bps)
P1	5'-aagcgatcctcctgcctca-3'	5'-cgtgtcctcagcgctggcgttcg-3'	KpnI	XholI	1796
P2	5'-aagcgatcctcctgcctca-3'	5'-cgtgtcctcagcgctggcgttcg-3'	SacI	XholI	1453
P3	5'-agtagatggcgctcaccttta-3'	5'-cgtgtcctcagcgctggcgttcg-3'	SacI	XholI	1024
P4	5'-gcacagtcgctttgagggcc-3'	5'-cgtgtcctcagcgctggcgttcg-3'	SacI	XholI	258
P5	5'-taggattcccgttggtatcg-3'	5'-cgtgtcctcagcgctggcgttcg-3'	SacI	XholI	197
P6	5'-gaaaacaggggcctctcac-3'	5'-cgtgtcctcagcgctggcgttcg-3'	SacI	XholI	139
P7	5'-cgggggagcgtagtctcg-3'	5'-cgtgtcctcagcgctggcgttcg-3'	SacI	XholI	100
P8	5'-cgaaaacaggggcctc tcacgtgacccctgcgcgctccc gcgggggagcgtagtctcg-3'	5'-cgagactacgctccccc gcgggagcgcgcaggggtca cgtgagaggcccctgttttcg-3'	SacI	XholI	39

### 3. Identification and mutation of AP-1-like binding sites in CD164 promoter

Sequence homology to the AP-1 consensus sequence (TGACTCA) was identified using TFBIND, which is a software programme for searching transcription factor binding sites. Mutation of AP-1-like sites and Ebox sites was performed by Stratagene's QuikChange II Site Directed Mutagenesis Kit.

Primers for distal AP-1 mutation are as follows: -

forward: 5'-CAG GGG CCT CTC ACG CTG TCC CTG CGC GCT CCC G-3';

reverse: 5'-CGG GAG CGC GCA GGG ACA GCG TGA GAG GCC CCT G-3'.

Primers for proximal AP-1 mutation were as follows: -

forward: 5'-CAG GGG ATT GAG GGG TCT GTT GAG CGT TGC GAG CCT TAG-3';

reverse: 5'-CTA AGG CTC GCA ACG CTC AAC AGA CCC CTC AAT CCC CTG-3'.

Primers for Ebox mutation: -

forward: 5'-CCG AAA ACA GGG GCC TCT AGA TTG ACC CCT GCG CGC TCC CGC GGG-3';

reverse: 5'-CCC GCG GGA GCG CGC AGG GGT CAA TCT AGA GGC CCC TGT TTT CGG-3';

Primers for Ebox deletion: -

forward: 5'-GCA CGC CGA AAA CAG GGG CCT CTA CCC CTG CGC GCT CCC GCG GGG-3';

reverse: 5'-CCC CGC GGG AGC GCG CAG GGG TAG AGG CCC CTG TTT TCG GCG TGC-3'.

### 4. Luciferase activity assay

Firefly (*Photinus pyralis*) luciferase is widely used as a reporter of promoter activities by cloning interested promoters to the upstream of the firefly luciferase coding gene. Nowadays, the dual-reporter assays, where the activities of firefly and Renilla (*Renilla reniformis*, also known as sea pansy) luciferases are measured sequentially from a single sample, are commonly used to improve experimental accuracy. The activity of the experimental reporter (firefly) is normalized by the activity of the internal control (Renilla). Cells were transfected with constructed reporter vectors simultaneously with phRL vector (Promega) containing cDNA coding Renilla luciferase; 2 ng of the latter was applied to each well in a 24-well plate. 48 hrs after transfection, cells were harvested with passive Lysis buffer, a component of the Dual-Luciferase Reporter (DLR™) Assay System (Promega), according to the manufacturer's instructions. An appropriate volume of cell lysate was added to a well of the F96 MicroWell™ Plates (NUNC, Roskilde, Denmark), followed by 25 μl of LARII. Firefly luciferase activities were measured with a luminometer (Tecan, Theale, UK). Renilla luciferase activities were also measured. Relative luciferase activity was represented by the ratio of firefly luciferase activity to renilla luciferase activity. Each experimental group included 3 repeats and data were shown as means.

### 5. Cell culture

In this work, HCT116 cells (cell line catalogues: ECACC 91091005) were exclusively used to study the role of CD164 in cancer development. All the cell lines were obtained from the European Collection of Cell Cultures (ECACC) and cultured in a CO_2 _incubator (Sanyo^®^). HCT116 and HT29 cell lines were cultured in DMEM and SW480, K562 in RPMI-1640 medium supplemented with penicillin G (100 U/mL; Sigma^®^), streptomycin (100 mg/mL; Sigma^®^) and 10% fetal calf serum (Gibco^®^). Cells were grown at 37°C in a humidified atmosphere of 5% CO_2 _and were routinely sub-cultured using 0.25% (w/v) trypsin-EDTA solution. For testing promoters' activity under hypoxia, cells were transiently transfected with pGL3P4 plasmids and cultured in DMEM without serum for 24 hours; then the cells were transferred into an Invivo_2 _400™ Hypoxia workstation (Ruskinn Technology Ltd) for different time courses.

### 6. Western blot

Protein used for western blotting was extracted with mRIPA buffer containing protease inhibitors (mRIPA, 50 mM Tris, pH 7.4, 100 mM NaCl, 1% Nonidet P-40, 0.5% deoxycholic acid, 0.1% SDS, Aprotinin 10 μg/ml, Leupeptin 10 μg/ml, PMSF 1 mM). Proteins were quantified using BCA^TM ^Protein Assay Kit (Pierce, Northumberland, UK). The Western Blot system was set up using a Biorad Bis-Tris Gel system, according to the manufacturer's instructions (Biorad, Hertfordshire, UK). Rabbit-anti-human HIF-1alpha antibody (Millipore) was prepared in 3% blocking buffer at a dilution of 1:1000. The primary antibody was incubated with the membrane at 4ºC overnight followed by a brief wash and incubation with secondary antibody for 1 hour at room temperature. Finally, peroxide and luminol solutions 1:1 (Pierce, UK) were added to cover the blot surface for five minutes at room temperature and the membrane was placed in a developing cassette.

### 7. Chromatin precipitation

Chromatin immunoprecipitation (ChIP) assay was performed using the Chromatin Immunoprecipitation Assay kit (EZ ChIP, Millipore) according to the manufacturer's instructions. Briefly, HCT116 cells were transfected with pCDNA3.1-HIF-1A. Forty-eight hours after transfection, the cells were then cross-linked by 1% formaldehyde for 10 min. The formaldehyde was quenched using 2 M glycine for 5 min. at room temperature before harvest. Cells were collected by centrifuging in PBS containing protease inhibitors and were lysed in SDS-lysis buffer. Soluble chromatin was prepared after sonication to an average DNA length of 200 to 1000 bp. Fragmented chromatin was immunoprecipitated overnight at 4°C on a rotating platform, using antibodies against HIF-1alpha (Millipore) together with Protein A/G Plus-Agarose. The agarose beads were washed, chromatin extracted and protein-DNA cross-links reversed. DNA was purified and was analyzed by PCR using the specific primers (5'-GCACAGTCGCTTTGAGGGCC-3'; 5'-CGTGTCCTCAGCGCTGGCGTTCG-3'). Normal immunoglobulin G (IgG) was used as a negative control. Anti-RNA Polymerase II was used as positive control. The total input was the supernatant from the no-antibody control.

### 8. Quantitative real-time polymerase chain reaction (qRT-PCR)

The total RNAs were isolated from cells with Trizol^® ^reagent (Invitrogen) and possible genomic DNA contamination were eliminated by treatment with RNase-free DNase 1 (Sigma) for 1 hr. The extracted RNAs were immediately reverse-transcribed using the SMART^TM ^MMLV RT Reverse Transcriptase CDNA Kit (Clontech). Real-time PCR was carried out on the iQ™5 Multicolor Real-Time PCR detection System (Bio-Rad) using the following specific primers: -

CD164 (5'-GTGCTGTCCGCGGACAAGAAC-3'; 5'-TGTGAACAATAGCTCTCATC-3');

GAPDH (5'-CGAGATCCCTCCAAAATCAA-3'; 5'-GGTGCTAAGCAGTTGGTGGT-3').

The PCR primer pairs were designed with Primer3 software. Primer specificity has been validated by BLAST analysis. Real-time PCR was performed using SYBR Green Master Mix (an asymmetrical cyanine dye by Promega) with Mg2+ concentration 6 mM, template cDNA and 0.5 μm of each primer, with RNA-negative and water controls. The qPCR cycle was 98°C for 2 min., 40 cycles of 95°C for 15 sec., 60°C for 30 sec. Final melt-curve analysis (60°-95°C) was included. The standard curve was produced with slopes at approximately -3.32 (~100% efficiency); PCR quantification used ΔΔct method for the CD164 against the GAPDH for normalization. The data are representative of the means of three experiments.

## Results

### Identification of CD164 promoter

**Figure 1:**
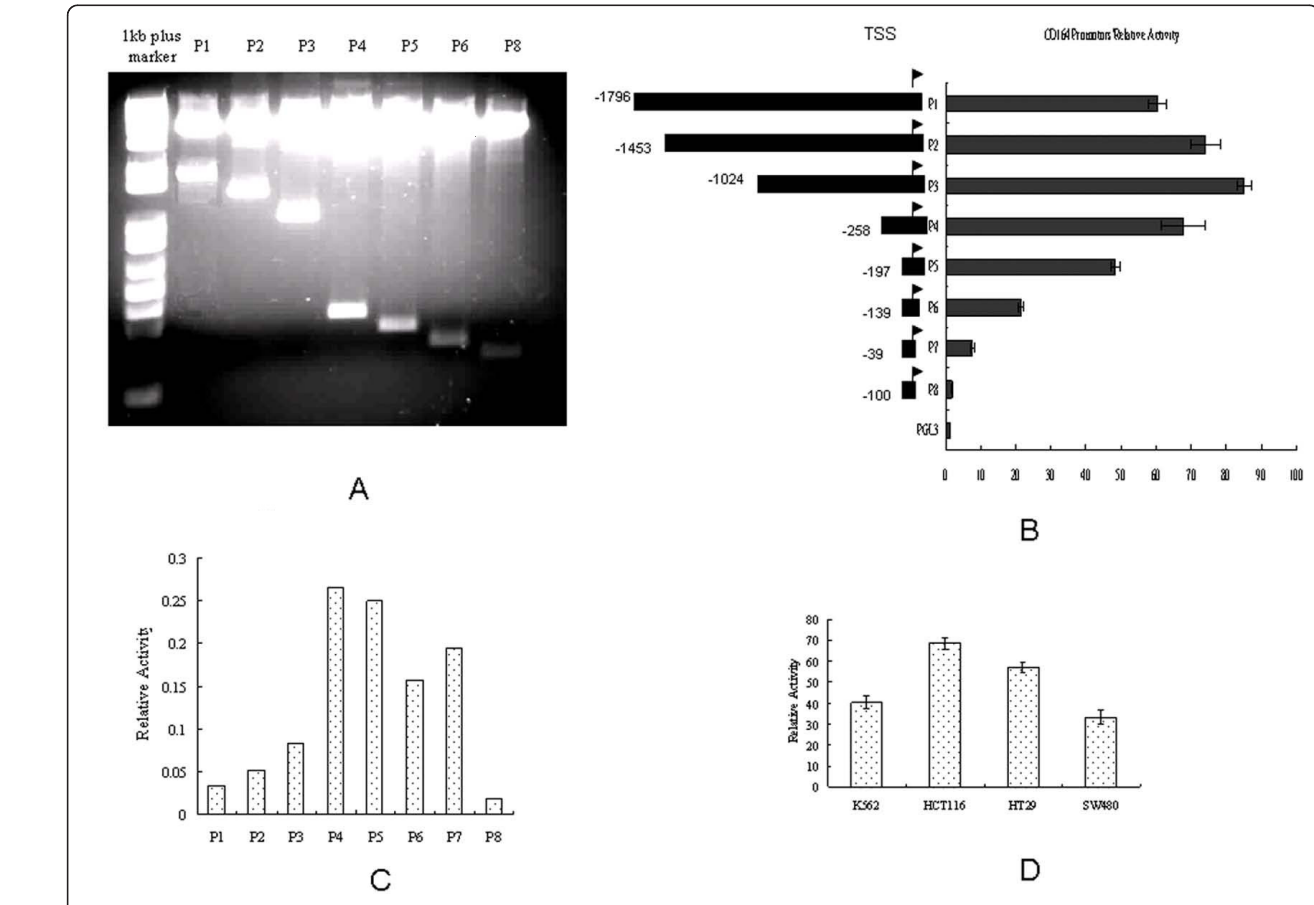
**Analysis of the 5'-flanking region of CD164 gene promoter**. (A) The PCR products of a series of 5'-deleted mutants of the CD164 gene promoter were subcloned to a pGL3 Luciferase reporter system at the SacI/XholI site, except P1.0 which used a KpnI/XhloI site to generate the CD164 promoter-pGL3 expression plasmid. (B) The 5'-deleted constructs were transfected into HCT116 cells with lipofectamine 2000. 48 hours after transfection, their luciferase activities were examined as described in Methods. Basic pGL3 plasmid without promoter was used as a control. Luciferase activity was normalized to the activity of renilla which were co-transfected as an internal control. The numbers represented the sites 5'-upstream of TSS. Deletion of the region from P2 to P3.2 resulted in a 75% reduction in basal promoter activity. The data bars represented the mean values of three experiments, each performed in triplicate. The error bars represented the standard errors. (C) Relative promoter's activity against promoter's length. P4 showed the optimal activity according to its length. These data suggested the existence of a minimal promoter region within the sequence. (D) Cell-type specificity of CD164 promoter activity. P4 activity was analyzed in different cell lines including K562, HCT116, HT29 and SW480. HCT116 cells showed the highest P4 activity.

Promoter 2.0 Prediction Server [14] was employed for predicting the CD164 promoter region. Promoter 2.0 predicts transcription start sites of vertebrate PolII promoters in DNA sequences. It can also be used to predict transcription factors that bind to the sequences in promoter regions, based on the theorems that are common to neural networks and genetic algorithms (http://www.cbs.dtu.dk/services/Promoter/). As first exon of the CD164 gene begins on 10418 [GenBank: AL359711], atgtcgcggc, a 3192 bp sequence at the 5' upstream of the transcription start site (TSS) was analyzed by Promoter 2.0 Prediction. The sequence of the putative promoter region of the gene is embedded at position -800 bp upstream of the TSS (Table 2). To determine the minimal promoter region, the 1.7 kb genomic sequence was subcloned in pGL3 basic upstream of the luciferase reporter gene. These 5'-deleted constructs pGL3 P1(-1796/1), pGL3 P2(-1153/1), pGL3 P3(-1024/1), pGL3 P4(-258/1), pGL3 P5 (-197/1), pGL3 P6(-139/1), pGL3 7(-135/-96) and pGL3 8(-100/1) were transfected to HCT116, as shown in Figure 1B. The primers amplifying the corresponding fragments were as shown in Methods. Renilla luciferase was used for normalization of firefly luciferase expression. The highest activity appeared in P3, in which the length of promoter is from -1024 upstream of the TSS (Figure 1B). P4 showed a lower level of activation than P3. However, balancing their length, P4 was recognized as the minimal promoter region for the CD164 gene (Figure 1C). Deletion of the region from P3 to P6 resulted in a 75% reduction in basal promoter activity. These data suggested the existence of a minimal promoter region within the sequence -258/1. P3 activity was analyzed in different cell lines including K562, HCT116, HT29 and SW480, of which HCT116 showed the highest P4 activity (Figure 1D).

**Table 2:** Prediction of the CD164 promoter using Promoter 2.0 Prediction Server

Position	Score	Likelihood
800	1.107	Highly likely prediction
2100	0.607	Marginal prediction
2800	0.588	Marginal prediction

The sequence of the putative promoter region of the gene is embedded at position -800 bp upstream of TSS

### In silico prediction of minimal promoter regions

**Figure 2:**
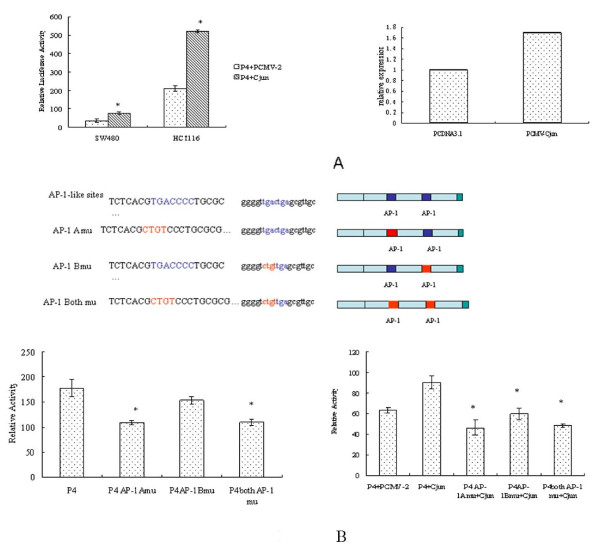
**Effects of c-jun expression on the transcriptional activation of CD164 promoter**. (A) SW480 and HCT116 cells were transiently co-transfected with the pGL3P4 (0.8 μg/μl) and pCMV-c-jun (0.8 μg/μl), luciferase activity of cell lysates was measured and normalized to renilla activity as internal **co**ntrol (left). Results represented the mean ± SD of three independent experiments. Exogenous c-jun significantly enhanced the CD164 expression, *p < 0.01, Student's t-test. The Real-time PCR were performed to investigate the upregulated expression CD164 at mRNA level in HCT116 (right). (B) Mutational analysis of AP-1-like sites on CD164 promoter fragment P4. Schematic representation of the pGL3P4 regions containing AP-1-like binding sequence and its mutants. pGL3P4, a wild-type construct containing 258 bps of the 5'-flanking sequence upstream of TSS; P4AP-1 Amu, a mutated pGL3P4 plasmid containing a mutated AP-1-like motif (CGCTGTCCC) at -119; P4AP-1 Bmu, a mutated pGL3P4 plasmid containing a mutated AP-1-like motif (GTCTGTTG) at -54; P4AP-1 Both mu, a mutated pGL3P4 plasmid containing two mutated AP-1-like motifs (CGCTGTCCC,GTCTGTTG) at both of -119 and -54. pGL3P4 and its mutants were transfected in HCT 116 cells respectively and assayed for luciferase activity The relative activities of wild-type P4 and AP-1-like mutated P4 mutants were normalized with renilla expression. P4AP-1 Amu and P4AP-1 Both mu resulted in significant decreases in luciferase activity when compared to pGL3P4, whereas no significant change of luciferase activity was observed in P4AP-1 Bmu.

The putative minimal promoter regions P4 (-258 upstream of the TSS) were analyzed with TFBIND (http://tfbind.hgc.jp/), a software program for searching transcription factor binding sites [15]. The P4 sequence contains two AP-1-like binding sites which are located at -119 bps (A, distal) and -54 bps (B, proximal) upstream of the TSS, respectively (Figure 2B). The results are shown in Table 3.

**Table 3:** Prediction of AP-1-like transcription factor binding sites on the P4 promoter

Access	ID	Score	Location	Strand	Consensus Sequence	Signal Sequence
M00174	V$AP1_Q6	0.818353	-119	(+)	NNTGACTCANN	CGTGACCCCTG
M00174	V$AP1_Q6	0.863106	-54	(+)	NNTGACTCANN	GTTGACTGAGC

AP-1-like binding sites (distal and proximal) were found

### Effects of C-Jun and HIF-1alpha on the P4 minimal promoter activity

C-Jun is a proto-oncogene. Its expression has been studied in many tumour types [16]. C-Jun, in combination with c-FOS, forms the AP-1 early response transcription factor. In order to determine the effect of the potential effects of C-Jun on minimal promoter P4 activity, we co-transfected HCT116 cells and SW480 cells with pCMV-c-jun or pCMV-2 empty vector and reporter vector pGL3 P4(-258/1). The C-Jun was able to increase the minimal promoter activity by about 50% in both cell lines (Figure 2A). The upregulation of CD164 by C-Jun at mRNA level was observed using real-time PCR (Figure 2A). To investigate whether the AP-1-like motifs play roles in CD164 gene expression, the wild-type pGL3 P4 construct or its mutants (P4AP-1 Amu, P4AP-1 Bmu, P4AP-1 *Both mu *pGL3 constructs) as shown in Figure 2B, were transfected into HCT116 cells. In HCT116 cells, the activities of P4AP-1 Amu and P4AP-1, *Both mu *mutants, were reduced by about 40% compared to pGL3P4 wild-type (Figure 2B). In contrast, there was no significant change of activity in P4AP-1 Bmu (p > 0.05 Student's t-test) compared to pGL3P4 wild-type. Significant difference (p < 0.05 Student's t-test) between P4AP-1 Amu and P4AP-1 Bmu showed the location of different responses to endogenous transcription factors. The distal AP-1-like motif which overlapped with HIF-1alpha binding sequence in the promoter was essential for CD164 transcription. The responses of AP-1-like motif to exogenous C-Jun were tested by co-transfected pCMV-C-Jun construct and the mutants. The mutants, especially P4AP-1 Amu and P4AP-1 *Both mu*, resulted in decreased activities compared to the wild-type. These data suggested that the AP-1-like motif is one of the main elements in the CD164 gene promoter. P4AP-1 Bmu, after treatment with exogenous C-Jun showed significantly different promoter activity, compared to the control. It seemed that AP-1-like site in -54 (proximal) was more sensitive to exogenous C-Jun than endogenous stimulatory factors.

**Figure 3:**
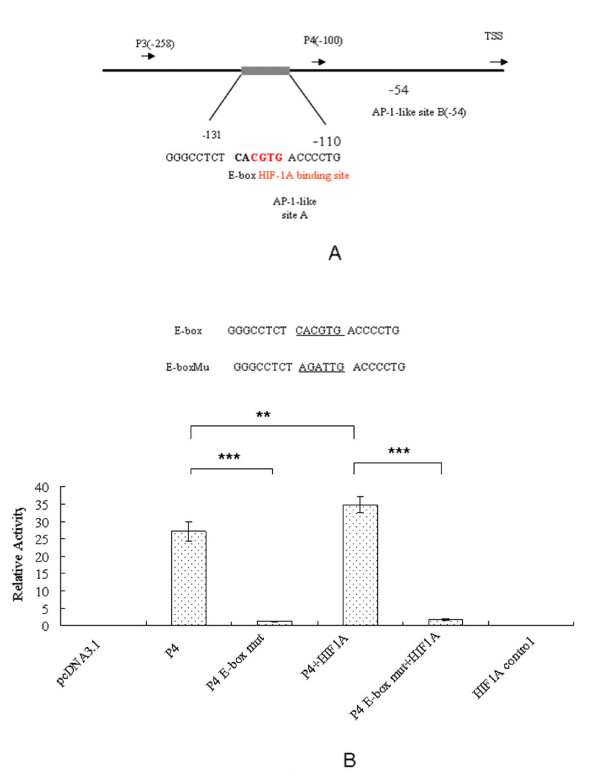
**The functions of overlapping E-box, HIF-1alpha and AP-1-like sequences within CD164 P4 promoter**. (A) Schematic presentation of the Overlapping HIF-1A/E-box/AP-1-like in the minimal promoter region of CD164. (B) Mutation analysis of the putative E-box on CD164 promoter P4 region. Constructs with mutation of the E-box in the promoter region were subcloned into the pGL3-basic plasmid and the HCT116 cells were transfected. Significant decrease of luciferase activity was found in the E-box mutation P4 mutant (p < 0.001, Student's t-test). Co-transfection of a HIF-1alpha expressing plasmid with pGL3P4 resulted in a significant increase in the promoter activity (p < 0.01, Student's t-test). To investigate the effect of HIF-1alpha through HIF-1alpha/E-box overlapping sequence, the pcDNA3.1- HIF-1alpha was co-transfection with PGL3P4 containing E-box mutation. For the normalized luciferase activity, a significant decrease of luciferase activity was found in the co-transfection with PGL3P4 containing E-box mutation compared to the HCT116 cells co-transfected with wild-type PGL3P4 (p < 0.001, student T-Test).

TFBIND also identified several other transcription factors which contain putative binding sites for transcription factors, several of which exactly match or are very similar to those already known, like the AP-1-like sites described above. It also revealed the presence of one HIF-1alpha motif (5_-RCGTG-3_) overlapping E-box (CACGTG) and distal AP-1-like binding sites (Figure 3A). To investigate the contribution of this E-box to the activity of the P4 region, constructs with mutation the E-box in the promoter region were subcloned into the pGL3-basic plasmids and used to transfect the HCT116 cells. For the normalized luciferase activity, a significant decrease of luciferase activity was found in the P4 containing E-box mutation compared to the HCT116 cells transfected with wild-type P4 (p < 0.001, student T-Test, Figure 3B). To determine whether HIF-1alpha mediates the increased activity of pGL3P4 in HCT116 cells, co-transfection of a HIF-1alpha plasmid with pGL3P4 was performed. Co-transfection of a HIF-1alpha expressing plasmid with pGL3P4 resulted in a significant increase in the promoter activity (p < 0.01, Student's t-test, Figure 3B). To investigate the effect of HIF-1alpha through HIF-1alpha/E-box overlapping sequence, the pcDNA3.1- HIF-1alpha was co-transfection with PGL3P4 containing E-box mutation. For the normalized luciferase activity, a significant decrease of luciferase activity was found in the co-transfection with PGL3P4 containing E-box mutation compared to the HCT116 cells co-transfected with wild-type PGL3P4 (p < 0.001, student T-Test, Figure 3B). It demonstrated that HIF-1alpha/E-box/AP-1-like sequences is crucial cis-regulatory elements of CD164 expression.

Hypoxia exists in the microenvironment of solid tumours and stem cells. In the present study, *in vitro *hypoxia treatment was carried out to examine if hypoxia mediates CD164 expression. The promoter activity was determined under hypoxic conditions and pGL3P4 plasmids were transiently transfected into the HCT116 cells. The cells were cultured in DMEM without serum for 24 hours, then transferred into gas-exchange chambers maintained at 37°C with 1% pO_2_.

**Figure 4:**
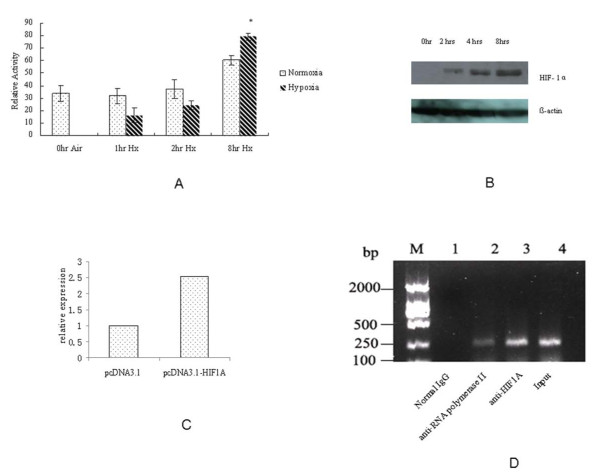
**Effects of hypoxia on CD164 expression**. (A) Hypoxia increased activity of CD164 promoter. HCT 116 cells were transfected with pGL3P4 plasmids and treated in hypoxia chamber. The normalized luciferase activities were shown. After 8 hours, the activity of pGL3P4 in hypoxia group was significantly higher than that of normal group (p < 0.05 student's t-test). No significant difference was found at 1hr and 2hrs. (B) HIF-1 alpha protein level was tested using western blot and there was an increase of HIF-1alpha protein expression in a time-dependent manner. (C) Real-time PCR was performed to investigate the upregulated expression CD164 at mRNA level in HCT116 after transfection of HIF-1 α. (D) Chromatin immunoprecipitation analysis of HIF-1alpha binding to the CD164 promoter. DNA released from the precipitated complexes was amplified by PCR. The PCR products were separated by agarose gel electrophoresis. Lane 1: IgG control; Lane 2: anti-RNA polymerase II; Lane 3: HIF-1alpha antibody pull-down; Lane 3:Input

Figure 4A shows that, compared to normal oxygen conditions, 2 hours of treatment of hypoxic conditions appear to depress pGL3P4 activity, although there is no significant difference. At 8 hours of hypoxic treatment, the activity of pGL3P4 was significantly higher than that of the normal oxygen level group (p < 0.05 Student's t-test). The upregulation of CD164 by HIF-1alpha at mRNA level was observed using real-time PCR (Figure 4C). The result suggested that hypoxia might be one of the factors upregulating CD164 gene expression in HCT116 cells. HIF-1alpha levels were therefore tested using western blot in the experiments described above. Figure 4B shows that HIF-1alpha protein expression was increased in a time-dependent manner in the hypoxic chamber.

### Binding of HIF-1alpha to PGL3P4 minimal promoter sequence

In order to confirm the binding of HIF-1alpha to the promoter of CD164, Chromatin immunoprecipitation (ChIP) assay was performed. We transfected HIF-1alpha plasmid to the HCT116 cells. The anti-HIF-1alpha was used to precipitate protein/chromatin complexes from sonicated samples. The complexes were then processed to DNA amplification. As shown in Figure 4D, it demonstrated the binding of HIF-1alpha to the minimal region of promoter of CD164.

## Discussion

A cis-regulatory element is a region of DNA or RNA near a gene required for proper spatiotemporal expression of that gene, often containing binding sites for transcription factors. The mechanisms by which CD164 expression is activated have been defined in this study. The different lengths of 5'-deleted mutant constructs of the CD164 showed different luciferase activities. The luciferase activities reached their peak in P2 which was 1024 bps upstream of the transcription star site and was 70-fold greater than that of the control group. The luciferase activity assays also indicated that the CD164 promoter had relative cell specificity, as its ability to activate luciferase was different in HCT116, K562, HT29 and SW480 cells. The PGL3P4 promoter had a proximal region of approximate 258 bases that increased the promoter activity by more than 50-fold and was recognized as the most effective promoter when compared to their sizes.

From the *in silico *analysis, the overlapping HIF-1alpha/E-box/AP-1-like sequences was found in the minimal region of CD164 promoter. C-jun and hypoxia upregulated the expression of CD164. Mutation of the overlapping sites resulted in reduced expression of CD164. Two AP-1-like sites (distal site and proximal site) on this proximal promoter region, of which distal site located in the overlapping sites, were identified by the *in silico *analysis. The consensus AP-1 (activator protein 1) recognition sequence is TGACTCA but many variations of this sequence (AP-1-like sites) were found in the promoter regions of target genes, including tyrosine hydroxylase [17], prodynorphin [18], proenkephalin [19] and glial fibrillary acidic protein [20]. Two putative AP-1-like sites in the CD164 gene PGL3P4 segment were found in the present study by bioinformatic analyses. The AP-1 transcription factor is a dimeric complex, and correlates with multi-stage tumor development, including tumor cell proliferation [21,22]; apoptosis [23,24]; tumor invasion and angiogenesis [25,26]. The main AP-1 proteins in mammalian cells are Jun and FOS. Endogenous and exogenous transcription factors influence the CD164 expression by binding to AP-1-like sites. In the condition without exogenous factors, the distal AP-1-like site, located at -119, which was supposed to bind endogenous factors including c-jun, may play a major role, as mutation of this site induced a 40% decrease of CD164 basal expression in HCT116 cells, whereas no significant effect was found by mutating the proximal AP-1-like site. Therefore, the distal AP-1-like site which is overlapped with HIF-1alpha/E-BOX sequence plays a more important role than the proximal AP-1-like site, which is located at -54 for endogenous factors. However, the proximal AP-1-like site was sensitive to exogenous c-jun It is likely that the CD164 expression in HCT116 cells is also controlled by both exogenous and endogenous factors through AP-1-like sites. CD164 may be a target gene in the AP-1 pathway and plays crucial roles in tumor development.

A number of studies have suggested that normal stem cells reside in "niches," which support and maintain the undifferentiated phenotype of the stem cells. These niches may be hypoxic and hypoxia may play roles in maintaining the stem cell phenotype. Under hypoxia, HIFs regulate a variety of pro-angiogenic and pro-glycolysis pathways. In solid cancers, regions of hypoxia are commonly present throughout the tissue. In this study, hypoxia treatment and the transfection of HIF-1alpha induced the expression of CD164. Our results and previous studies showed that eight hours of treatment in a hypoxia chamber resulted in the highest level of HIF-1alpha expression. The ChIP showed the binding of HIF-1alpha to the promoter of CD164. It demonstrated that the hypoxic treatment upregulated the expression of CD164 through HIF-1alpha binding the promoter of CD164.

## Conclusions

In conclusion, the results presented here have confirmed cis-Regulatory functions of overlapping HIF-1alpha/E-box/AP-1-like sequences of minimal promoter region of CD164. The research performed in this paper represents the combination of *in silico *biology and molecular biology. The elucidation of the cis-regulatory elements is useful in understanding CD164 functions in cancer cells.

## Competing interests

The authors declare that they have no competing interests.

## Authors' contributions

The work presented here was carried out in collaboration between all authors. JT, ZL, JX and GZ carried out the experiments. GL, JT, JX and GZ made contributions to design, analyze and interpret data. JX, FY and JT have been involved in drafting the manuscript. All the authors have given final approval of the manuscript to be published.
